# Power in Global Health: A Narrative Review, Relational Framework, and Systems-Based Analysis

**DOI:** 10.34172/ijhpm.9698

**Published:** 2026-08-02

**Authors:** Million Tesfaye Eshete, Goran Tomson, Ole Petter Ottersen, Tanja Tomson, Rhoda K. Wanyenze, Qingyue Meng, Eivind Engebretsen

**Affiliations:** ^1^Sustainable Health Unit, Medical Faculty, University of Oslo, Oslo, Norway; ^2^Department of Global Public Health, Karolinska Institutet, Stockholm, Sweden.; ^3^Oslo New University College, Oslo, Norway.; ^4^Institute of Basic Medical Sciences, University of Oslo, Oslo, Norway.; ^5^Department of Health Promoting Science, Sophiahemmet University Stockholm, Sweden.; ^6^Department of Disease Control and Environmental Health, School of Public Health, Makerere University, Kampala, Uganda.; ^7^China Center for Health Development Studies, Peking University, Beijing, China.

**Keywords:** Structural Power, Global Health Governance, Health Inequities, Power Asymmetry, Political Economy of Health

## Abstract

**Background:**

Power is routinely invoked to explain health inequities and governance failures in global health, yet existing work is conceptually fragmented, weakly connected to empirical analysis, and prone to what is referred to as the "power-as-resource fallacy": the conflation of power bases (what actors possess) with power relationships (how actors influence one another). This narrative review examines how power is conceptualized and operationalized in global health discourse, identifies systematic analytical gaps, and proposes a relational framework to address them.

**Methods:**

We conducted a narrative review of literature that explicitly engages with power in relation to global health, governance, or health systems. We synthesized the included texts thematically and, as a supplementary systems-thinking component, developed a causal loop diagram (CLD) based on a subset of quantitative studies to illustrate how different constellations of power interact with health outcomes over time.

**Results:**

In global health, power is pervasively invoked but rarely operationalized. We organized the reviewed texts into six thematic domains: (1) power as a determinant of health; (2) structural power (class, race, gender, geopolitical hierarchies); (3) power asymmetry; (4) power and governance for health; (5) power and health securitization; and (6) power constellations and health outcomes. Across these domains, we find that discussions of power remain largely qualitative and often stop short of clear operationalization. The CLD translates these insights into interacting feedback loops, illustrating how specific power constellations are associated with particular patterns of policy response and health outcomes, highlighting potential leverage points for governance reform.

**Conclusion:**

This review maps the existing knowledge on power in global health and provides an actionable framework for future research and governance design. It underscores that power is the fundamental thread connecting global political economy to local health inequities.

## Background

 Power asymmetries are not a recent feature of global health governance but a constitutive one. Imbalances in political, economic, sociocultural, and epistemic authority, shaping who sets the agenda, who benefits, and who bears the costs, have marked international and global health since its origins in colonial and tropical medicine, and were entrenched through the post-war institutional order, structural adjustment, and donor-driven aid.^1,2^

 These asymmetries are largely structural; they have persistently reproduced inequities and constrained progress toward global health justice.^3^ What distinguishes the present moment is not the emergence of power dynamics but their acute intensification. The upheavals following the return of the Trump administration in January 2025 illustrate how concentrated political power can rapidly reconfigure multilateralism and trust in science: the United States’ withdrawal from the World Health Organization (WHO),^4^ the termination of its funding, the spread of disinformation, and direct pressure on universities and scientific agencies have narrowed the policy space for health and turned political power against the financial and academic foundations of global health.^5^ These developments echo, in sharper form, the governance dysfunctions identified a decade earlier by the 2014 Lancet–University of Oslo (UiO) Commission on Global Governance for Health, which traced the political origins of health inequity to five failures: a democratic deficit in global governance, inadequate policy space for health in the face of powerful economic actors, institutional inertia and stickiness, missing or weak institutions for cross-sector governance, and accountability gaps.^6^ These dysfunctions have since deepened rather than resolved; examining how the field has theorized power therefore provides essential context for responding to current challenges.

 Whatever definition of global health one adopts,^7-10^ the field cannot achieve its goals through scientific and technological advancement alone. Biomedical interventions operate within complex socio-economic and political contexts that determine their reach, accessibility, and equity. Consequently, addressing health inequities requires looking beyond technical solutions to confront the relational and political power dynamics that shape human societies.

 In his foreword to Paul Farmer’s Pathologies of Power, Sen boldly declared, “Power prevents the sharing of different opportunities, resulting in health inequity.”^11^ Similarly, in the words of Paul Farmer himself, “Careful study reveals that state power has been responsible for most human rights violations, and most of these violations are embedded in “structural violence”—the social and economic inequities that determine who will be at risk for assaults and who will be protected.”^12^ Framing the inescapability of power in the discourse and practice of global health requires a deeper examination of the term “power.”

 This presents a significant challenge, as power is universally recognized as an elusive concept to define. Whether power qualifies as an “essentially contested” concept remains subject to debate. For policy questions—such as how to achieve health equity given the explanatory role of power—simply acknowledging that power is contested provides little practical guidance. Some definitions are more analytically useful than others, offering clearer pathways for diagnosis and intervention. We note that the relationship between power and health is bidirectional: power shapes health outcomes, but health crises can also erode state capacity and reshape power relations. This review focuses on the power-to-health direction as its primary analytical scope; the reverse pathway is considered in the discussion.

###  Epistemological and Ontological Issues

 Across the scholarly literature, authors consistently acknowledge how difficult power is to define; it is named far more often than it is studied, and described as “elusive,” “hopelessly abstract,” “conceptually fluid,” and “amorphous.”^13-15^ Accounts oscillate between treating power as a property or possession, debated as an excess or deficit and invoked to attribute health crises to too little or too much power among key actors,^16,17^ and treating it as a relationship between actors in the political-science tradition.^18,19^ They also distinguish normatively between “power over,” cast as repressive and zero-sum,^20^ and the more “consensual,” “power to,” “power with,” and “power within.”^21,22^

 A further distinction concerns the *domain* in which power operates rather than its valence.Normative power denotes the capacity to change, by a voluntary act, the existence, content, or application of a norm—thereby creating, modifying, or extinguishing rights, duties, and obligations, as in promising, contracting, or legislating.^23^ Epistemic power, by contrast, operates in the domain of knowledge and belief: it is the capacity to shape what counts as legitimate knowledge and who is recognized as a credible knower, with its asymmetries manifesting as the credibility deficits and exclusions theorized in accounts of epistemic injustice.^24,25^

 Some scholars link these various forms of power to global health inequities.^26^ Despite this diversity of conceptualizations, the notion of power as a resource appears to underpin much of the global health literature. A recent review, for instance, defined power asymmetries as the “unequal distribution of resources (such as money, knowledge, or political authority) among actors.”^27^ This perspective is often used to describe how a lack of resources can lead to limited influence and poor health outcomes. However, following Baldwin, this definition can be seen as the “power-as-resource fallacy.”^28^ Considering the multidimensionality of power is critical to this end. Importantly, the resource view and the relational view are better understood as analytically distinguishable emphases along a continuum than as mutually exclusive positions: even when power is described as a “resource,” its significance only becomes apparent in relational contexts, since resources confer power only insofar as they create leverage over or with others.

####  Working Definition

 Having surveyed these definitions, we adopt a bounded working position for this review. Drawing on Baldwin’s^28^ multidimensional framework, we define power as the capacity of an actor to influence outcomes in a specified domain, exercised through identifiable means, at specifiable costs, and always in relation to other actors. This definition is deliberately relational: it requires that any claim about power specify who exercises it, over or with whom, regarding what, and at what cost. It is also deliberately multidimensional: it recognizes that power operates through coercive, institutional, structural, and productive mechanisms (following Barnett and Duvall),^29^ which are analytically distinguishable but empirically bundled in any given political configuration. We do not claim to resolve all definitional debates; rather, we commit to a position precise enough to organize the synthesis that follows and to identify where the literature falls short.

 This narrative review aims to synthesize how power is understood and operationalized in global health discourse, providing a comprehensive overview that critically examines current approaches and identifies key conceptual and analytical gaps. We have consciously prioritized breadth over depth, mapping the landscape of power in global health rather than providing fine-grained analysis of every subtopic. This choice reflects our assessment that the field’s primary deficit is not a shortage of detailed case studies but a lack of conceptual integration across them.

 We aim to: (1) identify the key conceptualizations of power used in global health discourse; (2) examine the mechanisms and pathways through which power influences health inequities; (3) critically assess the strengths and limitations of current theoretical and analytical approaches to power; (4) propose a more robust framework for analyzing power to advance global health equity; and (5) outline the implications of this framework for the future of global health governance and new multilateralism.

## Methods

###  Design

 We conducted a narrative review to map and interpret how power is conceptualized and operationalized in global health, an approach suited for summarizing heterogeneous, theory-rich literature. Searches were not time limited. We queried Medline, Embase, CINAHL, Scopus, Web of Science, PsycINFO, and ProQuest. We also used The Lens and Google Scholar to surface seminal contributions, considering citation counts alongside relevance. Search strings were customized per database, and definitions of the broader conceptual terms used throughout, including global health, global health governance, and health inequities, are provided as supplementary material (See Supplementary file 1). In collaboration with expert librarians at Karolinska Institutet, we performed forward and backward citation chasing from seed articles to expand coverage. The initial searches of bibliographic databases/registers and targeted grey literature were run on October 11, 2023 (searched from inception; no retrospective date limits; no language restrictions at the search stage). We used the structured database search as an entry point. We then iterated via close reading and citation tracking; following a hermeneutic systematic review logic previously applied in a WHO/Europe Health Evidence Network synthesis report.^30^ Following screening and full-text assessment, we conducted iterative citation tracking (backward/forward snowballing) from included studies to identify additional records and relevant research traditions. Citation tracking and iterative identification were conducted on a rolling basis until we concluded that theoretical saturation had been reached. Consequently, the evidence set was closed for synthesis on June 30, 2025, after which no further records were added. We did not aim for exhaustive retrieval, and we did not treat the database search as a comprehensive enumeration of the literature; instead, we sought conceptual saturation had been reached; defined here as the point at which new sources ceased to yield novel frameworks, mechanisms, or typologies linking power to health outcomes.

 To complement our qualitative synthesis, we conducted a post-hoc bibliometric analysis of the 1144 *un-screened* records retrieved from the Web of Science. We isolated this subset because its standardized metadata allows for reliable natural language processing and disciplinary mapping. This analysis served two purposes. First, a term-frequency analysis was used to quantify the extent to which power is analytically specified versus merely invoked in the literature. Second, because “power” is fundamentally a political science and international relations construct, we conducted a journal-level mapping to verify that our search successfully penetrated these disciplines. This step was taken to ensure our search net captured canonical political science and international relations theories of power for engagement in the qualitative synthesis. The findings of this mapping are detailed in the results section.

###  Eligibility

 Eligible sources included peer-reviewed articles, commentaries, theses, and relevant book chapters. We excluded conference abstracts, blog posts, and non-English items. To be included in the final analysis, a text was required to meet all three of the following criteria. Specifically, it had to: (1) explicitly define or theorize “power” as an explanatory variable; whether structural, institutional, relational, or discursive—rather than using the term as a colloquial descriptor or background context; (2) focus on health inequities (systematic, avoidable differences in health outcomes) rather than general health indicators alone; and (3) articulate a theoretical or empirical link between the power dynamic identified and the health equity outcome examined. We excluded studies where “power” was used only as a colloquial descriptor. Texts that invoked power only as a rhetorical device, without specifying the actors, mechanisms, or domains involved, were excluded. This review focuses on the power → health direction as its primary analytical scope; the reverse pathway is addressed as a limitation and a direction for future research in the discussion.

 To model how these dimensions interact, we developed a causal loop diagram (CLD) from a purposively selected subset of quantitative studies (multi-country panel studies, systematic reviews, and reviews of reviews) that operationalize governance, regime type, and welfare through established indices: authoritarianism, autocratisation, democratic participation, decommodification, austerity, and citizen empowerment.

###  Analysis

 From the included texts (Figure 1) we extracted approximately 200 single-spaced pages of verbatim excerpts—definitions, descriptions, and debates on power in global health. We used a narrative, inductive approach, reading the data without predefined categories to identify recurring ideas and patterns. After the data-driven phase, we undertook a deductive, theory-informed reading to test explanatory adequacy and resolve inconsistencies. This stage ensured theoretical fit and coherence by mobilizing well-established frameworks from political theory and international relations and other disciplines (detailed in the respective section) to interpret the inductively derived themes. We coded sentences/paragraphs as discrete units, iteratively grouping similar codes into candidate themes. Coding and theme development followed established guidance for thematic analysis and qualitative synthesis.^31-33^

**Figure 1:**
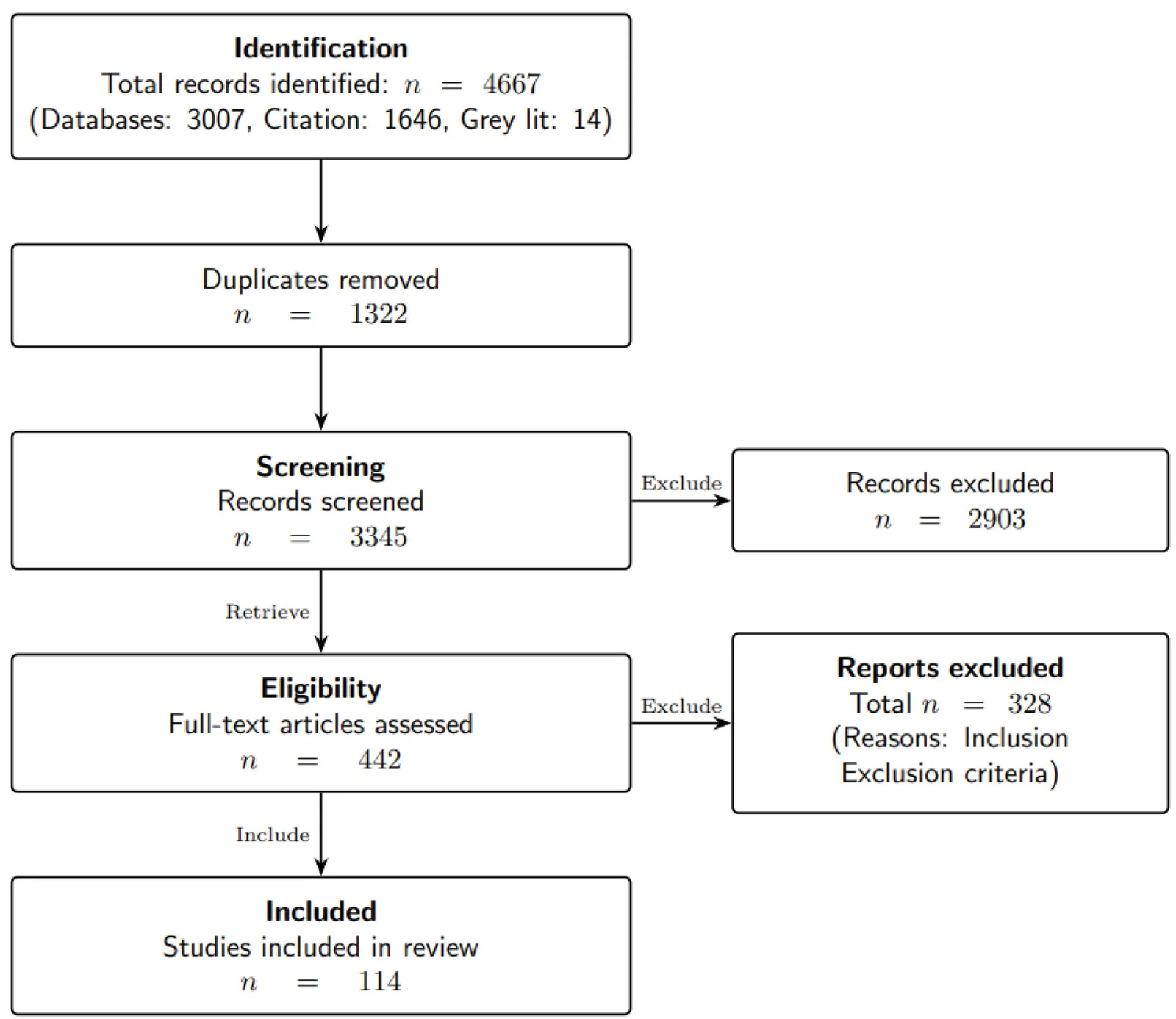
Study Identification, Screening, and Inclusion Flow (PRISMA-Style Flow Diagram With Iterative Citation Tracking).^34^ Abbreviation: PRISMA, Preferred Reporting Items for Systematic reviews and Meta-Analyses.

 To characterize the broader discourse landscape, we also conducted a bibliometric term-frequency analysis of the 1144 abstracts retrieved from the Web of Science search. Using natural language processing, we measured the prevalence of analytically specified power concepts (eg, “structural power,” “epistemic power,” and “compulsory power”) against descriptive or thematic terminology (eg, “power dynamics,” “power relations,” and “governance”), characterizing the field in the literature’s own language without judging individual studies. We also classified the disciplinary distribution of source journals.

 In parallel, to move beyond conceptual debates about what power “is,” we analyzed a purposively selected subset of quantitative studies (systematic reviews, reviews of reviews, and multi-country or nationwide panel studies) using a systems-thinking approach, chosen because they operationalize power or link specific power constellations to observable global-health outcomes. Drawing on both the narrative themes and these quantitative findings, we developed the CLD and examined it for plausible reinforcing and balancing feedbacks, following established system-dynamics practice.^35^ Model development followed best practices of iterative refinement, alignment with the evidence base, and parsimony.^36^ The narrative review is therefore organized into six thematic categories that incorporate the CLD results.

## Results

###  Landscape of the Discourse

 Before the thematic synthesis, we characterize the landscape of the literature on power and global health from the Web of Science search (N = 1934 records, of which 1144 contained abstracts suitable for analysis). The literature spans multiple disciplines (N = 1073 articles and reviews): global health, health policy and systems, public health and epidemiology, political science and international relations, social sciences and humanities, and biomedical and clinical fields, with nearly half in multidisciplinary venues (Figure 2). Notably, 62 records from 45 political science and international relations journals confirm that the search reached beyond health-specific literature.

**Figure 2:**
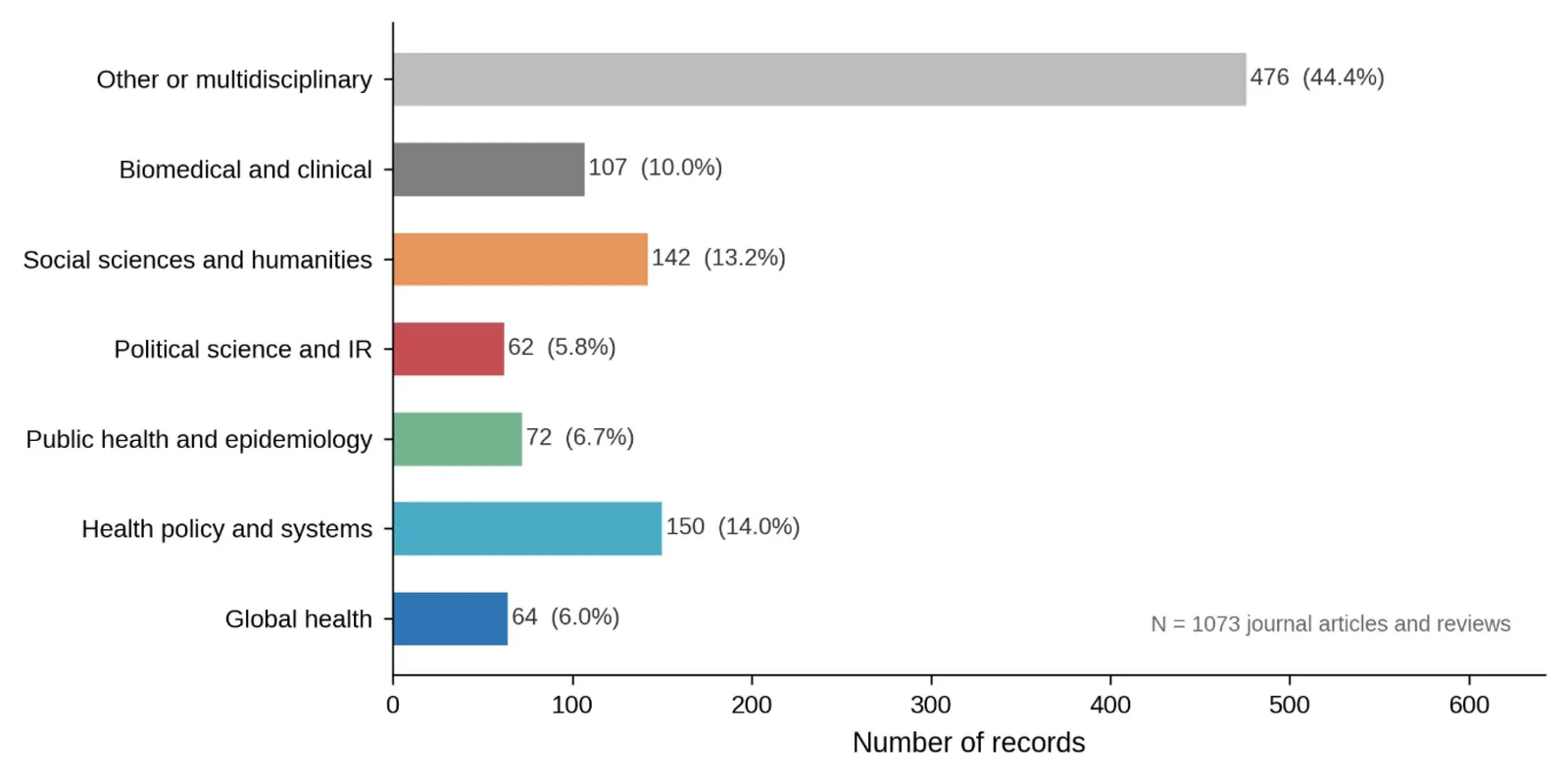
Disciplinary Profile of Source Journals in the Web of Science Search Results (N = 1073 journal publications). Abbreviation: IR, international relations.

 A term-frequency analysis of the 1144 abstracts reveals a striking pattern we term the operationalization gap (Figure 3). Descriptive or thematic terms dominate: “governance” appears in 14.1% of abstracts, “power relations” in 8.0%, “power asymmetry” in 6.8%, and “power dynamics” in 5.6%. By contrast, analytically specified concepts are nearly absent: “structural power” in 0.7%, “institutional power” in 0.7%, “epistemic power” in 0.7%, “productive power” in 0.5%, and “compulsory power” in 0.1%. This contrast, between the frequent rhetorical invocation of power (5.6%–14%) and the rare use of specific analytical typologies (<1%), supports a long-standing critique: in global health, power is frequently named but rarely theorized. The operationalization gap frames the thematic synthesis that follows, in which we examine what the literature says about power and, equally important, what analytical tools it uses or fails to use. Table 1 lists the various definitions of power extracted from these disciplines. Table 2 summarizes the dimensions of power that must be considered when assessing whether one actor holds power over another, drawing on frameworks described in detail elsewhere.^28,37^

**Figure 3:**
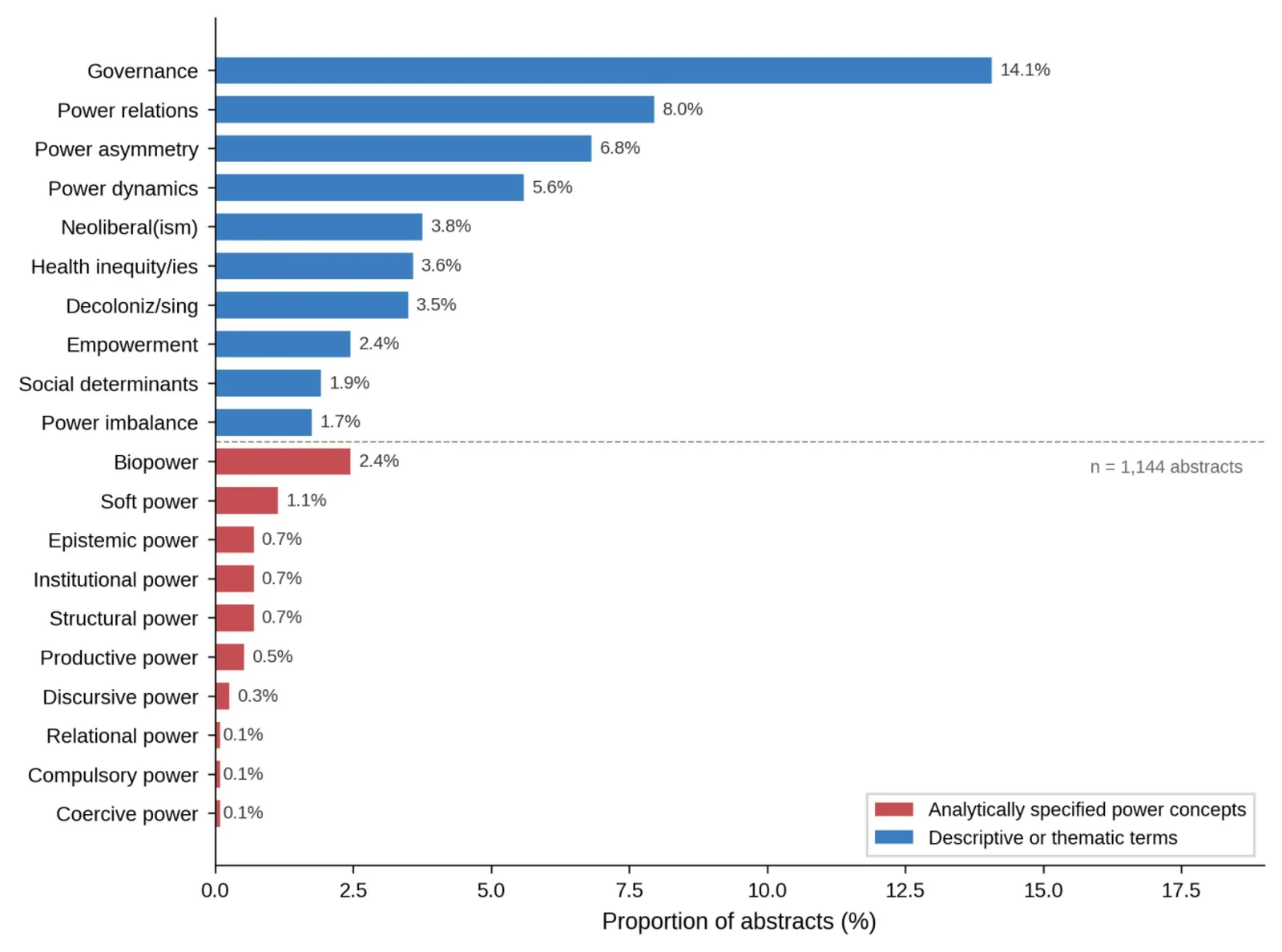
Frequency of Descriptive Versus Analytically Specified Power Terminology in Abstracts (n = 1144).

**Table 1:** Definitions of Power From Different School of Thought

**Reference**	**Definition/Statement About Power**	**Discipline**	**Implications**
^38^	"But power is also exercised when A devotes his energies to creating or reinforcing social and political values and institutional practices that limit the scope of the political process to public consideration of only those issues which are comparatively innocuous to A."	Political science	The subtle and indirect forms of power through agenda-setting, institutional practices, and mobilization of bias. Often referred to as the "second face of power."
^19^	"Power is the probability that one actor within a social relationship will be in a position to carry out his own will despite resistance."	Sociology/political economy	Power as a relational concept involving the ability to enforce one's will despite opposition.
^29^	"Whereas institutional power focuses on the constraints on interest-seeking action, structural power concerns the determination of social capacities and interests."	International relations	Given the interchanging use of the terms (institutional and structural), distinctions are often overlapping.
^39^	"A has more power than B, if A achieves many intended effects and B only a few."	Philosophy	Power in the intended direction (intentional and intended). Effectiveness is relative to the achievement of intended outcomes.
^37^	"A has power over B to the extent that he can get B to do something that B would not otherwise do."	Political science	Power in the direction of intended effect and normatively neutral.
^40^	"A may exercise power over B by getting him to do what he does not want to do, but he also exercises power over him by influencing, shaping or determining his very wants."	Political sociology	Power that manufactures preferences and needs. The strongest form of power (Lukes' third face). Submission does not equal the absence of power.
^41^	Structural power is “…the power to shape and determine the structures of the global political economy within which other states, their political institutions, their economic enterprises and (not least) their scientists and other professional people have to operate."	International political economy	Unintended power; power that is impersonal and acknowledged by the virtue of A’s presence.
^42^	"Power is 'The ability of one actor to alter the decisions made and/or welfare experienced by another actor relative to the choices that would have been made and/or welfare that would have been experienced had the first actor not existed or acted.'"	Economics	Power in the direction of both intended and unintended effects.
^43^	"Power shall refer to the ability to tell other people what to do with some degree of certainty that they will do it."	Economics	The focus is on the abstractness of power. The power concept here does not appear at first. Supreme exercise of power is at play. B might comply without resistance because A first secured that B’s options are limited.
^44^	"It is asymmetries in interdependence that are most likely to provide sources of influence for actors in their dealings with one another."	International relations	Instead of resources, asymmetric interdependence is the source of power. The most misunderstood concept of power as far as the term “power asymmetry” is concerned.
^45^	Kenneth Boulding identifies three types of power: destructive, productive, and integrative.	Economics	Premium is placed on moral power. All other forms of power are short-term.
^46^	"Power corresponds to the human ability not just to act but to act in concert... Power is never the property of an individual; it belongs to a group and remains in existence only so long as the group keeps together."	Political philosophy	A relational concept of power. Distinguishes between force, violence, and power.
^47^	"Law exerts a powerful influence on health by structuring, perpetuating, and mediating the social determinants of health... Law can be a powerful tool for securing and advancing health and equity."	Law	Power as legitimate political authority.
^48^	“…structural social power if the structure of the interactions is consistently biased in [favor of one group]…and, therefore, so are their outcomes…” “...conventional social power if the outcomes of interactions between agents [of a different group members] conform to some discriminatory evolutionary equilibrium even though the interactions are structurally symmetrical.”	Evolutionary game theory (economics)	Radical solidarity on behalf of the unfortunate is the only viable intervention.
^49^	“...soft power is more than just persuasion or the ability to move people by argument... It is also the ability to attract, and attraction often leads to acquiescence. Simply put, in behavioral terms, soft power is attractive power."	International relations	Soft power is attraction power. It is best understood within Lukes' third-dimensional view of power.
^50^	"An analysis of the power relations affecting biomedicine addresses questions like who has power, how this power is delegated, expressed, and manifested in the social relations of healthcare, and what are the principal contradictions and arenas of struggle and resistance."	Critical medical anthropology	Power that operates in biomedicine.

**Table 2:** Dimensions of Power Relevant to Assessing Whether A Holds Power in Relation to B

Dimensions of Power*	**Definition**	**Example**
Base of power	A has a greater power base if A possesses more economic assets, social networks, prestige, etc, compared to B.	A multinational corporation (A) has more financial resources and global influence than a local small business owner (B).
Means of power (conversion factors)	Conversion factors that transform A's power base into actions influencing B's responses. These means can be abstract (skill, charm, charisma) or concrete (threats, promises, symbols, etc).	A charismatic clinician-influencer (A) turns reputation into action via social proof, parasocial ties, and simple calls-to-action that lower effort for followers (B).
Scope of power (the range of actions A can legitimately take)	The range of actions A can legitimately take to influence B, such as passing laws, vetoing decisions, or enforcing regulations.	A government (A) can enact policies affecting the entire population (B). An international body like the WHO or UN Security Council (A) can impose sanctions or provide aid to nations (B).
Amount of power (Probability shift)	The net increase in the likelihood of B performing actions recommended by A due to A's influence.	If a regulator (A) threatens fines, and a company (B) complies with regulations to avoid penalties, A has demonstrated power over B.
Extension of power	A's ability to reach or influence multiple individuals or groups (Bs) simultaneously.	A social media platform (A) can affect the behavior of millions of users (B) through algorithm changes or content policies.
Opportunity costs	The relative cost of compliance vs. resistance. A has more power if it is cheap for A to act but costly for B to resist.	A major supplier (A) can easily find new buyers, but a small retailer (B) relies heavily on A's products. B is more likely to comply with A's terms to avoid business losses.

Abbreviations: WHO, World Health Organization; UN, United Nations.

###  Power as Determinant of Health 

 The literature reviewed consistently identifies power as a fundamental determinant of health outcomes, though it operationalizes the concept in different ways and emphasizes different causal pathways. The WHO Commission on the Social Determinants of Health defines power inequalities as the main cause of health inequities.^51,52^ Here, the Commission on the Social Determinants of Health frames “power inequalities” as a force of social stratification—determining one’s position within a hierarchy. In contrast, the Lancet-UiO Commission frames “power asymmetry” as a failure of global governance—a symptom of “bad politics” where unequal influence shapes the rules of the game.^6^ Despite this distinction between stratification and governance, both reports converge on a shared conclusion that power is a systemic process that structures relationships and distributes resources. Notably, neither report provides a specific definition of power, nor do they clearly operationalize the concept.

###  Power and the Notion of Structure

 Across the literature we reviewed, structural power is the dominant lens for explaining how power produces health inequities.^14,53-55^ The term “structural” is used in three analytically distinct but related ways in this literature: as rule-setting capacity in international relations (following Krasner and Strange); as institutionalized racial hierarchy within states (following Michener and others in public health); and as welfare state architecture and political economy (following Beckfield and Krieger in social epidemiology). These uses share a common core—all treat power as operating through durable arrangements that shape what actors can do before any individual interaction occurs—but they operate at different levels of analysis and implicate different intervention points. In social epidemiology, scholars explicitly situate power within political–economic and governance arrangements, showing how institutionalized priorities and rules shape the distribution of risks and resources—for instance, through welfare regimes, neoliberal restructuring, or political incorporation (the extent to which marginalized groups are formally included in political institutions and decision-making processes).^55^ Within public health, authors increasingly define structural racism itself as a power relation—“institutionally rooted” and process-oriented—that organizes access to housing, services, and political voice, thereby structuring exposure and vulnerability. This reframing shifts the focus from individual racial differences to the institutional systems and practices that produce inequality.^14,56^ This perspective aligns with the concept of the “second face of power,” where power is exercised not through open conflict but through the “mobilization of bias”—the norms, institutions, and procedures that covertly structure benefits and risks without overt resistance.^38^

 Global health literature often draws on international relations and political economy to define and operationalize structural power as the capacity to shape the context in which choices are made. Krasner terms this *meta-power*—the ability to structure decision environments—and because “structure” spans institutional, economic, political, and social arrangements, operational definitions vary.^57^ Similarly, Strange defines structural power as the power to shape and determine the structures of the global political economy, operationalized across the domains of security, production, finance, and knowledge.^41^ Her account also allows power to operate without deliberate intent, as rules and market architectures generate unintended yet consequential effects.^41,58^ A dependency perspective, following Caporaso, reframes structural power as asymmetric vulnerability: actors with few affordable exit options are more easily influenced, so dependence itself functions as a value-allocating process that sustains power relations.^59^ Epistemic power—the capacity to define what counts as valid knowledge and who counts as a knower, thereby shaping agenda-setting, rule-setting, and gatekeeping in global health— is also sometimes framed as structural power.^24,60,61^

 These lenses explain different mechanisms relevant to global health. Krasner^57^ directs attention to how powerful actors set the rules that govern aid, trade, intellectual property (IP), and security—thereby structuring downstream population health. Strange^58^ foregrounds how authority is distributed across multiple structures and actors (diffusion of power where corporations shaping production/finance/knowledge), explaining why power in health often appears diffuse rather than centralized. Dependency perspectives^59^ emphasize asymmetrical vulnerabilities between and within countries that reproduce inequities in capacity, policy space, and exposure. Hence, in public health power should be treated as relational, institutional, and systemic, not merely a resource possessed by individuals—an orientation reflected in newer frameworks such as Health Power Resources theory.^22^

###  Power Asymmetry 

 A prominent theme in the global health literature is power asymmetry^[1]^ as a root cause of health inequity.^6,65-68^ Though rarely defined explicitly, it is illustrated through exemplification across contexts. Scholars have extensively documented how power imbalances shape academic partnerships and research collaborations between high-income countries (HICs) and low- and middle-income countries (LMICs).^1,3,69-74^ A significant portion of research conducted in LMICs is published without any LMIC-based authors, and most such publications are authored exclusively by individuals from HICs.^75^ Power asymmetry is also evident in the governance of global health organizations: reports note that 75% of board seats in influential global health organizations are occupied by HIC nationals,^76^ and the Global Health 50/50 report finds that over 80% of leadership roles are held by HIC nationals, with most organizations headquartered in Europe and North America.^77^

 Authors also analyze power asymmetry through a wider socioeconomic and political lens. Transnational health-harmful commodity corporations influence health policy through lobbying and legal threats^78^ and act as direct perpetrators of structural violence through extractive practices that states often fail to regulate. Researchers document vast economic outflows from the Global South to the Global North, with billions lost to unequal exchange and debt service,^79^ limiting the fiscal space for LMICs to invest in essential public services; in 2022 alone, LMICs paid $443.5 billion in debt service, with “devastating human consequences.”^80^

 The Trade-Related Intellectual Property Rights (TRIPS) agreement is a frequently cited case. Authors argue that IP-exploiting trade agreements are a direct product of asymmetric bargaining power,^81^ and read TRIPS as structural power in Strange’s sense: influence arising not from any single actor’s coercion but from the architecture of the regime itself, the capacity to shape the rules within which others must operate.^41^ Negotiated under asymmetric bargaining, its effects, higher medicine costs, are borne disproportionately by LMICs.^81,82^ The access-to-antiretrovirals struggle in sub-Saharan Africa is the paradigmatic illustration: HIC-headquartered firms used patent rules to maintain prices that excluded millions living with HIV,^83^ 68 until the use of TRIPS flexibilities (compulsory licensing, parallel importation) by Brazil, India, Thailand, and South Africa showed that such arrangements, while powerful, are not immutable when affected actors mobilize collectively.^84^

 Finally, some contend these asymmetries are maintained through the structure of global governance institutions: the International Monetary Fund, World Bank, and World Trade Organization are dominated by HICs, particularly the Group of 7, which hold a disproportionate share of voting power and decision-making control, reinforcing existing imbalances and shaping global health policy to favor powerful interests.^82^

###  Power and Governance for Health 

 In this section, “governance” refers primarily to macro-political governance (the institutional and ideological arrangements through which political power is exercised at national and global levels) rather than to health system governance in the narrower operational sense. The evidence reviewed here examines how the distribution of power across different governance configurations shapes health outcomes and inequities. The crucial distinction is between institutions (formal structures) and power (the relational capacity exercised through and around them): institutions are not power, but power operates through institutions by enabling certain actors to set agendas, veto decisions, and distribute resources.

 A review by Ciccone et al highlights a significant positive relationship between various forms of governance and health outcomes, whether directly or indirectly, by moderating other systemic factors that affect health.^85^ Many authors identify democracy as critical for an equitable distribution of power. Research across 102 countries has shown a strong association between the degree of democracy and lower mortality, with effective governance and corruption control reducing famine mortality.^86^ Higher levels of democracy also mitigate the negative health impacts of unfavorable economic and trade policies, such as those related to exports and multinational corporations.^51^ A study of 170 countries further confirms that democracies are more likely to maintain universal health coverage even during economic recessions, when access to care is most critical.^87^

 The literature also indicates that certain governance models, specifically social-democratic welfare regimes, distribute power more equally. “Left-of-centre political traditions” are linked to better child and maternal health outcomes, particularly in LMICs, through welfare-state measures and progressive policies aimed at the social conditions of deprived populations.^88^ Such regimes are reported to increase individuals’ “resisting resources,” empowering them to avoid deprivation. Several systematic reviews and quantitative studies confirm that high public spending, universal health coverage, participatory policy-making, and high-quality governance (rule of law, low corruption) are associated with better population health.^89-91^

 In Brazil, Gonçalves found that municipalities adopting participatory budgeting, which transfers a share of resource-allocation decisions from elected officials to citizen assemblies, experienced significant reductions in infant mortality over 1990–2004, an empirical case of health gains following the redistribution of agenda-setting power.^92^ Conversely, authoritarian regimes and neoliberal ones (limited public spending, extensive privatization) are consistently associated with poor population health (Figure 4).^55,93^

**Figure 4:**
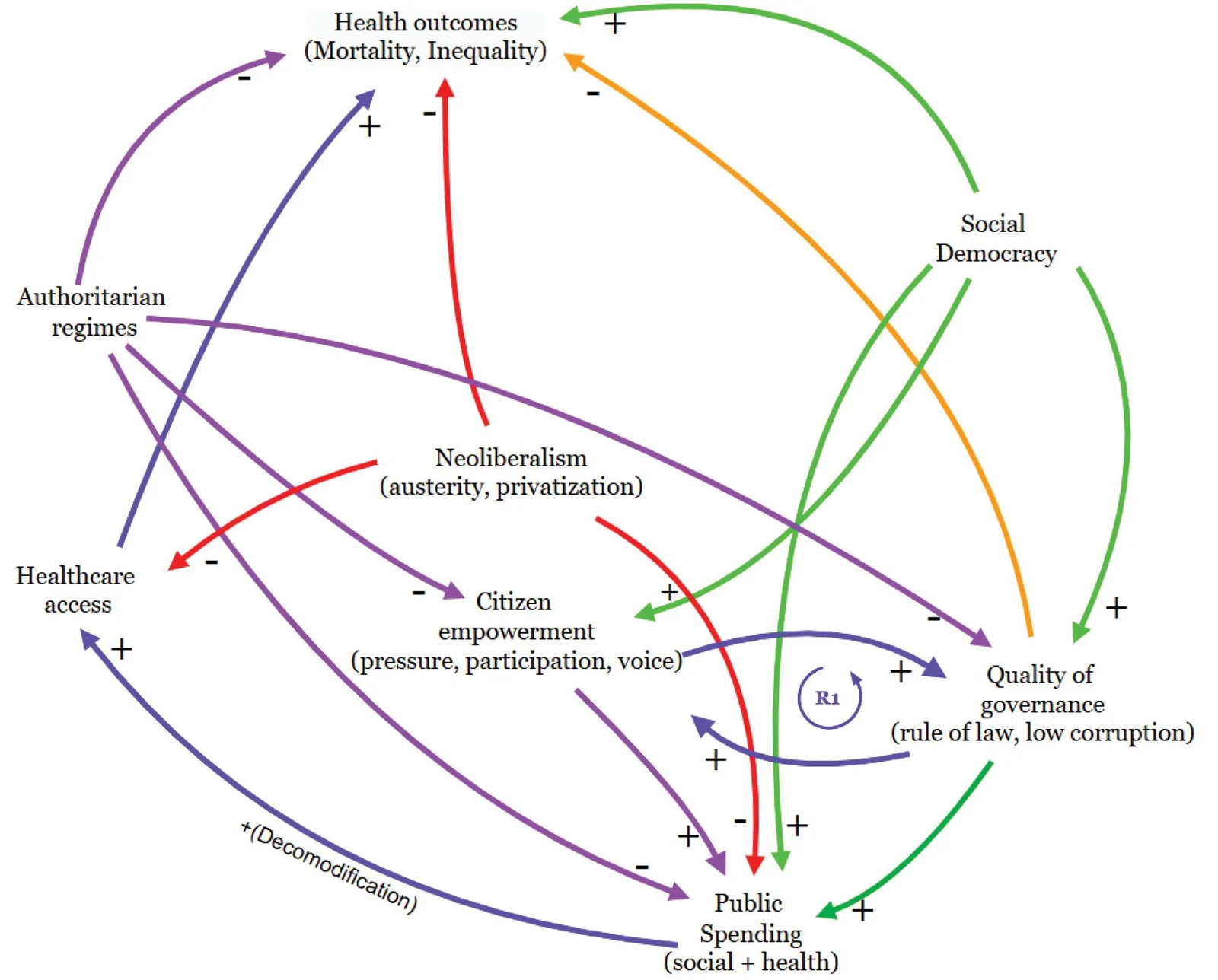
Power Constellations and Health: A Causal Loop Diagram. Arrows represent hypothesized causal influences between variables. A plus sign (+) indicates that the connected variables move in the same direction (an increase in one tends to increase the other); a minus sign (−) indicates an opposite effect. Colors group related pathways: purple, macro-political power relations (authoritarianism, citizen empowerment dynamics); red, neoliberal and austerity pathways; green/orange, social-democratic and quality of governance pathways; blue, welfare and public health system channels (public spending, decommodification, healthcare access). R_1_ marks a reinforcing feedback loop connecting citizen voice, governance, and spending. “Health outcomes” refers to population mortality and health inequalities.

 The relationship is not deterministic, however. Some authoritarian states, have achieved substantial gains in infant mortality and life expectancy through centralized resource mobilization, targeted public health campaigns, and state-directed investment in health infrastructure.^94^ Concentrated executive power, when directed at health, can produce measurable improvements, even if such gains often prove fragile and unevenly distributed.

###  Power and Health Securitization 

 A significant theme in the literature is the ongoing debate about health security and its complex relationship with power.^95-98^ Some authors indicate that this concept must be understood within the context of global geopolitical shifts, particularly in the post-World War II era. As the threat of traditional warfare evolved, health became a new instrument of state power, often used in foreign policy, national security, and diplomacy.^26,99^ This transformation, sometimes characterized as “high politics,” has been seen as an opportunity for health to become a “powerful currency” in international relations.^100^ However, there is considerable debate about the nature and true purpose of this shift. As Fidler explains, the rise of health in foreign policy is not always driven by a genuine commitment to “health for all.”^101^ The dominant theme in the reviewed literature is that global health, when used for diplomatic, security, and foreign policy purposes, is often less concerned with broad public health goals and more with the security and economic interests of HICs.^102,103^

 It is argued that this approach prioritizes issues perceived as direct threats to powerful nations—such as pandemics—while neglecting arguably more serious problems in LMICs, like diarrheal diseases.^99^ Authors suggest that this securitization of health legitimizes and reproduces existing power imbalances in the international system, allowing inequalities to persist.^103^ This dynamic is evident in responses to past crises, such as the Ebola outbreak, where the “global” response was criticized for being reactive and driven by whether the “rich and powerful feel threatened.”^104^

 To understand health securitization as an exercise of power, it is necessary to draw on the Copenhagen School’s securitization framework.^105^ In this framework, securitization is not an objective condition but a discursive act: a securitizing actor (typically a state or institutional authority) frames a referent object (in this case, population health or a specific disease threat) as facing an existential threat, and in doing so claims legitimacy for extraordinary measures that bypass normal political deliberation. The act succeeds only if a relevant audience accepts the framing. Applied to global health, this framework reveals securitization as an exercise of discursive and agenda-setting power: the capacity to define what counts as a “crisis,” to determine which health threats warrant exceptional response, and to shift decision-making from inclusive democratic arenas into securitized spaces governed by urgency and executive authority.^106^ The power at stake is not merely the power to respond to threats, but the prior power to determine which threats are recognized and whose security is prioritized.^104^ This reinforces the finding that despite calls from global health actors to use health assistance for purely humanitarian ends, the reality is that such initiatives often serve political and economic objectives.^98,103^

###  Power Constellations and Health

 This section traces how these dimensions interact within political power constellations (the macro-level configurations of governance, ideology, and institutional design that bundle multiple power mechanisms) and models their joint effects on health through the CLD. The included quantitative studies, alongside the resulting CLD, highlight how different constellations of political power shape health through recurrent feedbacks between institutions, spending, and access. Across multi-country panel studies, systematic reviews, and reviews of reviews, social-democratic arrangements and higher quality of governance were consistently associated with greater public social and health spending, stronger decommodification of welfare, and better healthcare access, which related to improved population health and reduced health inequalities (Table 3).

**Table 3:** Evidence Underpinning Causal Loop Diagram Links

**References**	**Methodology**	**Outcomes (Level)**	**Outcomes (Equity)**	**Direction of Causality** **(A Influences B, in the Direction of B)**	**Effect/Polarity**
^93,107^	Analytical review; review of reviews; research synthesis	LE; amenable mortality; MMR; IMR	SII/RII for LE, MMR, IMR; CI for coverage	Social democracy → Public/social spending	+
^108^	MC (170 countries, 1990-2019); systematic analysis (GBD study)	UHC Service Coverage Index (SDG 3.8.1); effective coverage	Financial protection (catastrophic OOP, SDG 3.8.2); tracer equity	Public/social spending → UHC (service coverage & protection)	+
^89-91,109^	MC (149 countries); systematic review of reviews; research synthesis	U5MR; LE; amenable mortality	SII/RII for U5MR/LE by wealth/education	Quality of governance (rule of law, low corruption) → UHC/outcomes	+
^92^	Nationwide sample (Brazil, 1990–2004)	IMR; sanitation/PHC coverage	SII/RII for IMR; gaps by municipality SES	Citizen empowerment (participation/pb) → governance & allocation → outcomes	+
^110^	Comparative analysis (29 countries) using OECD/WHO data	Amenable mortality; UHC SCI; OOP share	CI of service use (pro-poor); gradient in OOP burden	Decommodification (↓marketization) → access/UHC	+
^111^	Scoping review (EU-28) regarding the Great Recession	Public & health spend; treatable mortality; IMR/LE	SII/RII for IMR/LE; unmet-need gradients	Neoliberalism/austerity → public/social spending	−
^55,112^	MC (25 post-communist countries, 1989–2002)	Adult working-age mortality; cause-specific (CVD, injury)	SII/RII by education/wealth	Neoliberalism/privatization (rapid mass) → health outcomes	−
^108^	MC (204 countries, 1990–2019); GBD 2019 analysis	Amenable mortality; LE; MMR; IMR	SII/RII or CI on tracer coverage; CHE distribution	UHC coverage → outcomes	+
^94^	MC; GBD 2016 and other sources	Cause-specific adult mortality; LE; Government health spend	UHC SCI disparities; inequality in outcomes	Authoritarianism → governance/coverage/outcomes	−

Abbreviations: MC, multi-country (cross-national); UHC, universal health coverage; SCI, Service Coverage Index (SDG 3.8.1); SDG, Sustainable Development Goal; LE, life expectancy; U5MR, under-5 mortality rate; IMR, Infant mortality rate; MMR, Maternal mortality ratio; OOP, out-of-pocket payment; CHE, catastrophic health expenditure; SII/RII, Slope/Relative Index of Inequality (absolute vs relative gradients); CI, Concentration Index (including Erreygers-corrected); QoG, quality of governance; PB, participatory budgeting; PHC, primary healthcare; SES, socio-economic status; WHO, World Health Organization; OECD, Organisation for Economic Co-operation and Development; EU, European Union; GBD, Global Burden of Disease; CVD, cardiovascular disease. Effective coverage: Quality-adjusted coverage. Financial protection: SDG 3.8.2 indicators (OOP, CHE). Amenable mortality: Deaths avoidable with timely care. Key: →: Direction of influence in the CLD. Effect (+): Variables move in the same direction. Effect (−): Variables move in opposite directions. ↑/↓: Observed change in indicator relative to baseline.

 These relationships are mutually reinforcing: effective, less corrupt governance supports redistributive public spending, which strengthens health and social systems and consolidates accountable governance; at the center is a reinforcing loop between citizen empowerment and quality of governance (R₁). In contrast, neoliberal reforms (austerity, privatization) and authoritarian regimes were linked to lower citizen empowerment, constrained public spending, weaker public health systems, and poorer access to care, feeding into worse health outcomes and widening inequalities. The CLD thus visualizes how regime type, governance quality, public spending, and citizen voice mutually reinforce either more equitable or more exclusionary health trajectories.

 Consequently, the CLD maps power as a relational dynamic produced by the tension between macro-political regimes, economic paradigms, and civic agency. Rather than treating “social democracy” as a monolithic source of power, the diagram unbundles its empirical pathways, showing how democratic political architectures establish the conditions for decommodification, which in turn drives the public health spending necessary to reduce mortality.^113^ The CLD deliberately operates at the level of macro-political constellations (democracy, authoritarianism, social democracy, neoliberalism) rather than annotating individual arrows with discrete power types. When a social-democratic arrangement improves health through higher public spending, several mechanisms operate inseparably along that pathway: citizen agenda-setting through elections, institutional power through parliamentary structures, and normative power through solidaristic policy culture. Because these cannot be disaggregated at the macro-political level at which quantitative evidence exists, annotating arrows with discrete types would impose false precision and commit the very error this paper diagnoses: treating analytically distinguishable forms of power as empirically separable variables.

## Discussion

 Eleven years after the Lancet-UiO Commission on Global Governance for Health located the “deep causes of health inequity” not in technical failings but in fairness, justice, and political power asymmetries,^6^ assessments still find that theories of power remain underdeveloped and underutilized in global health research and practice.^25^ Amid a rapidly shifting geopolitical landscape, remapping these foundations is increasingly urgent. This review confirms a shift in global health discourse: power is no longer a background variable but a determinant of health in its own right. Yet this recognition has not translated into analytical precision. Our bibliometric analysis reveals a field that invokes power pervasively but operationalizes it rarely: “power dynamics” and “power relations” appear in 5%–8% of abstracts, while analytically specified concepts (structural, institutional, epistemic power) each appear in fewer than 1%.

 Two analytical habits contribute to this gap. The first is the tendency to treat power as a possession rather than a relationship. The reviewed literature frequently oscillates between attributing health crises to a deficit of power among certain actors and attributing them to an excess concentration of power in others. Both framings presuppose that power is a stock (something held in greater or lesser quantities) rather than a flow between actors in specific domains. Resources matter, and the relational view does not deny this: the point is analytical priority, since resources confer power only insofar as they generate leverage over or with others in a given context.

 This is the long-recognized *power-as-resource fallacy*—what Morriss called the “vehicle fallacy” and what Lasswell and Kaplan anticipated in distinguishing “base values” from power itself,^114,115^ and Baldwin’s “paradox of unrealized power” captures the same intuition: an actor with overwhelming material assets can fail to translate those assets into outcomes when relational and institutional channels are absent.^62^ For global health, the implication is concrete. Donor wealth does not automatically produce policy influence; epistemic standing does not automatically translate into agenda-setting authority; institutional position does not automatically yield rule-making power. Each translation requires specifiable mechanisms, and our review suggests these mechanisms are precisely what the literature most often leaves unspecified.

 The second habit is the dominance of “power over” framings at the expense of collaborative alternatives. While terms such as “power asymmetry” and “power imbalance” correctly describe relational leverage and vulnerability between actors, they are frequently misapplied in the literature to imply a zero-sum, possession-based logic. This matters practically: if the field’s analytical vocabulary defaults to domination, its prescriptive vocabulary will default to resistance rather than also exploring solidarity, coalition-building, and institutional redesign as power strategies. This changes what counts as a diagnosis. A field that asks who holds power will reach for redistribution; a field that asks through what mechanism does A actually shift B’s options will additionally consider rule-change, coalition formation, and the contestation of legitimating discourses. Both moves are needed, but the second has been undertheorized.

###  Power and Health Inequities: Mechanisms and Pathways

 Power imbalances are frequently framed as a root cause of health inequities, operating through pathways from global to local levels; tackling inequity, as the WHO Social Determinants and Lancet–UiO Commissions concluded, requires tackling the inequitable distribution of power.^6,52^ We identified several robust pathways.

 First, political ideology and regime type are decisive upstream determinants: social-democratic and high-quality-governance configurations are associated with better and more equitable health outcomes, while neoliberal restructuring and authoritarian concentration are associated with worse ones.^116^ The explanatory variable, however, is not the regime *label* but the underlying *power configuration*—whether mechanisms of accountability, redistribution, and citizen agenda-setting are institutionalized. Authoritarian states can deliver aggregate health gains through concentrated executive power, but without accountability mechanisms those gains tend to be fragile, unevenly distributed, and reversible.^94^ The relevant question for global health is therefore not “which regime type?” but “which power mechanisms produce sustained equity?”

 Second, the participation–governance loop (R₁ in Figure 4) operates as a *power-generating* rather than merely power-distributing mechanism—the distinctive feature of “power with.” Collective participation does not redistribute a fixed quantity of influence but produces additional governance. The Brazilian participatory budgeting evidence is instructive precisely because the mechanism is identifiable; citizen assemblies acquired genuine agenda-setting authority over allocation, and the health gains followed from this shift in *who decides*, not merely from increased spending.^92^ Where civic space is constricted, the loop inverts, with disempowerment and poor governance reinforcing one another. Identifying this loop empirically matters because it specifies an intervention point (institutionalized citizen agenda-setting) that is invisible to analyses framed solely in terms of resource redistribution.

 Third, at the global level, agenda-setting power shapes which health problems are recognized as crises and therefore funded, through what has been termed the health securitization paradigm. Dominant states and donors define what counts as a “global health crisis” in ways that reflect their security interests, channelling funding toward perceived threats (pandemics, bioterrorism) while neglecting endemic burdens (childhood diarrhea, maternal mortality), so that billions flow to pandemic preparedness and biodefense even as primary care in low-income settings remains underfunded.^95,97,99,104^ Securitization operates through a productive mechanism: it does not coerce LMIC governments but constitutes the vocabulary in which legitimate priorities are articulated, so the reproduction of imbalance is a feature of the mechanism, not incidental, which is why securitized architectures prove resilient even when their inequities are acknowledged, since contesting them requires reconstituting the frame, not only reallocating within it.^106,117^

 The mechanism at stake shapes the appropriate responses.Where compulsory power is exercised through coercion or conditionality, legal, regulatory, and rights-based strategies are most relevant: where structural power works through the rules of trade, IP, or financial regimes, reform requires coalition-building and institutional redesign rather than resisting individual actors. Where epistemic power imbalances reproduce themselves through publishing norms and research funding architectures, the response lies in equitable partnership frameworks, editorial reform, and sustained capacity investment in LMIC institutions. Correctly diagnosing the mechanism of influence—its base, means, scope, and cost—is a precondition for selecting an effective counter strategy.

###  Strengths and Weaknesses of Existing Approaches

 Global health scholarship draws on a rich arsenal of social and political theory, sensitizing the field to the different faces of power (visible, hidden, invisible), to levels from the global to the interpersonal, and to power exercised through both coercion and consent. This pluralism is a genuine strength; its weakness is application. Few empirical studies deploy any framework systematically, power is often invoked in passing or illustrated anecdotally, and terms such as “power asymmetry,” “empowerment,” and “influence” are used interchangeably across studies. The field is rich in conceptual tools but lacks a shared, operational method—the gap the following framework is designed to close.

###  Toward a Robust Framework for Analyzing Power and Health Equity

 The synthesis to this point has been descriptive; what follows is the more adjudicative analytic stance we propose. The field’s predominant approach has been typological: classifying power into named forms and mapping which actors hold which type. Moon’s expanded typology represents one of the most comprehensive modern attempts, categorizing power in global governance into eight forms: physical, economic, structural, institutional, moral, discursive, expert, and network,^68^ itself building on Barnett and Duvall’s four-part scheme (compulsory, institutional, structural, productive).^29^ But such frameworks largely define power by what actors possess rather than by how influence operates between them—the power-as-resource fallacy, and its corollary the “fungibility trap,” the mistaken assumption that power resources convert across domains like currency.^28,62,115,118^

 We therefore distinguish three things the typologies conflate: the base of power (what an actor holds), the channel through which it travels, and the mechanism by which it operates. Most of Moon’s forms (physical, economic, moral, expert, discursive, network) name bases or channels. Once these are set aside, the distinct mechanisms reduce to Barnett and Duvall’s four,^18,37,62^ which fall into two registers. The interactional register works through relations between pre-constituted actors: compulsory power (direct influence of A over B) and institutional power (indirect influence through the rules and procedures of institutions). The constitutive register works through relations that define actors and meanings before any interaction: structural power (constituting actors’ positions and capacities) and productive power (constituting subjects, meanings, and legitimacy). The distinction matters because the registers are not analysed with the same tools—and conflating them is what lets “structural power” pass through an analysis uncorrected. “Moral power,” for instance, is not a mechanism but a base that may be exercised compulsorily (shaming), institutionally (treaty obligations), or productively (defining legitimacy).

 The power bases that the global health literature often emphasize like moral authority, institutional position, epistemic standing, material resources, network centrality; are not competing “types” of power but entries in the first row of Baldwin’s multidimensional framework (Table 2). Identifying the base is only the first step; the analytical work lies in specifying how that base translates into actual influence over specific actors regarding specific outcomes, through what means, in what domain, and at what cost.

 To make this concrete, consider the TRIPS/antiretrovirals case through Baldwin’s columns. The *base* of pharmaceutical-firm power was a combination of patent assets, research and development capacity, and privileged access to HIC trade negotiators. The *means* was not direct coercion of LMIC governments but the embedding of patent rules into a binding multilateral regime that made non-compliance costly. The *scope* was the rule-set governing pharmaceutical pricing and parallel importation. The *domain* was global trade law, deliberately chosen over the WHO precisely because the latter offered LMICs more institutional voice. The *opportunity-cost asymmetry* was decisive: contesting TRIPS individually was prohibitively costly for any single LMIC, whereas maintaining the regime was nearly costless for firms. The eventual counter-mobilization by Brazil, India, Thailand, and (in the form of credible threat) South Africa shifted only one column (the opportunity-cost asymmetry) by aggregating LMIC bargaining weight through coalitional action and by using the political cost of being seen to deny impeding access to antiretroviral therapy as a counter-resource.^81,83^ The structural arrangement itself was not dismantled; what changed was the cost of enforcing it against coordinated resistance. This is what mechanism-specific analysis adds: it tells us not only that power was asymmetric, but precisely which column was contestable and through what means.

 Pulling these threads together, we propose a five-step heuristic for power analysis: (1) identify actors A and B and the specific outcome—vague claims about “donor power” rarely survive the demand to specify who influences whom about what; (2) specify the mechanism and its register—interactional (compulsory, institutional) or constitutive (structural, productive); (3) for interactional mechanisms, map the base, means, scope, and domain; for constitutive mechanisms, ask what the arrangement makes thinkable and legitimate; (4) identify the opportunity-cost asymmetry—where compliance is cheap for A and resistance costly for B, change requires shifting the cost structure through coalition, rule-change, or counter-discourse; and (5) trace feedbacks, since power relations rarely operate as one-shot exchanges. This gives researchers and decision-makers a shared, mechanism-specific, evidence-anchored protocol bridging the rhetorical invocation of power and its operational study.

###  Implications for Global Health Governance and Multilateralism

 Current governance arrangements often suffer from pronounced asymmetries – between wealthier and poorer nations, donor agencies and implementers, or corporate interests and public health priorities—and identifying them pinpoints single points of failure and where reform is needed. The Pandemic Accord negotiations illustrate this: agenda-setting power over treaty language, pathogen-sharing, and benefit-sharing remained concentrated among a few high-income states and pharmaceutical interests even as the accord nominally sought equity.^119^ A power-aware approach would treat such asymmetries as explicit design considerations (equity safeguards, rotating leadership, independent accountability) rather than background conditions.

 Understanding power also enables more resilient structures. Institutions that institutionalize “power with” through civil-society inclusion, transparency, and accountability are not only fairer but more robust to ideological swings, because they rest on broad ownership rather than the benevolence of a few; governance built on hegemonic leadership or ad-hoc coalitions may achieve quick wins but crumbles when political winds shift. Efforts like the Pandemic Treaty or health-financing compacts should therefore build in power-sharing so that rules cannot be easily rewritten by the most powerful in a crisis. Institutional design is necessary but not sufficient, however: power also operates through informal channels, norms, and relationships, and even well-designed institutions can be captured if these go unaddressed. A power-aware approach must combine formal reform with ongoing attention to who participates, whose knowledge counts, and whose interests are served in practice.

###  Limitations

 Some limitations apply. As a narrative review prioritizing conceptual saturation over exhaustive retrieval, we may have missed relevant studies; while our search of seven databases and citation tracking captured the canonical political-science and global-health literature, applied work in niche venues may be underrepresented. The bibliometric analysis characterizes broad discursive patterns from abstract terminology, highlighting field-level operationalization gaps rather than study-level depth. Finally, the review focuses on the power→health pathway; the reverse dynamic (how health crises reshape power^120^) is plausible but empirically underdeveloped.

## Acknowledgements

 The authors thank the expert librarians at Karolinska Institutet for their assistance with the search strategy. The authors also thank the Lancet-UiO Commission on Global Governance for Health Secretariat at SUSTAINIT, including Anja Annesdatter Riiser, for administrative and logistical support.

## Disclosure of artificial intelligence (AI) use

 The initial draft of this manuscript was written entirely by the authors without the assistance of AI tools. During manuscript preparation, the authors used Claude/ChatGPT strictly for English language proofreading and grammatical review. The authors reviewed and edited all AI-assisted suggestions and take full responsibility for the content of the final manuscript.

## Ethical issues

 As a review of publicly available literature, this study did not involve human participants, medical records, or identifiable data. Consequently, institutional review board approval was not required.

## Conflicts of interest

 Authors declare that they have no conflicts of interest.

## Endnotes


^[1]^ Despite its popularity in global health, the prevailing use of “power asymmetry” is often critiqued in international relations and political economy. Following Keohane and Nye^44^ and Baldwin,^62^ power in these contexts is understood as asymmetrical interdependence: relational power defined not by resource disparities but by vulnerability (the asymmetric costs each party would bear in disrupting or exiting the relationship). The term also obscures that even unequal relations remain bidirectional^63^ and that power operates through anticipated reactions, without observable exercise.^64^ Because “power asymmetry” remains the dominant nomenclature in the global health literature reviewed here, we retain the term as operationalized by each author; where used in our own analysis, it denotes asymmetrical interdependence.

## Supplementary files


Supplementary file 1. Conceptual Definitions and Search Documentation.
