# Supplementary material for: Power in Global Health: A Narrative Review, Relational Framework, and Systems-Based Analysis
Source: Int J Health Policy Manag. 2026 Aug 2;15:9698. doi: 10.34172/ijhpm.9698 (PMC13770244; doi:10.34172/ijhpm.9698)
Supplement: Supplementary file 1 [file ijhpm-15-9698-s001.pdf]

Article title: Power in Global Health: A Narrative Review, Relational Framework, and Systems-Based Analysis

Journal name: International Journal of Health Policy and Management (IJHPM)

Authors' information: Million Tesfaye Eshete[1]*, Goran Tomson[2], Ole Petter Ottersen[1,2,3,4], Tanja Tomson[5], Rhoda K. Wanyenze[6], Qingyue Meng[7], Eivind Engebretsen[1]

[1]Sustainable Health Unit, Medical Faculty, University of Oslo, Oslo, Norway.

[2]Department of Global Public Health, Karolinska Institutet, Stockholm, Sweden.

[3]Oslo New University College, Oslo, Norway.

[4]Institute of Basic Medical Sciences, University of Oslo, Oslo, Norway.

[5]Department of Health Promoting Science, Sophiahemmet University Stockholm, Sweden. [6]Department of Disease Control and Environmental Health, School of Public Health, Makerere University, Kampala, Uganda.

[7]China Center for Health Development Studies, Peking University, Beijing, China.

***Correspondence to:** Million Tesfaye Eshete; Email: mtesfaye1@gmail.com

Citation: Eshete MT, Tomson G, Ottersen OP, et al. Power in global health: a narrative review, relational framework, and systems-based analysis. Int J Health Policy Manag. 2026;15:9698. doi:10.34172/ijhpm.9698

Supplementary file 1. Conceptual Definitions and Search Documentation

Text S1.

Core conceptual references used to define "Global Health," "Global Health Diplomacy," "Global Health Governance," and Related Constructs

Global Health. While several systematic reviews and working definitions exist, clear consensus remains elusive. We define global health as "an area for study, research, and practice that places a priority on improving health and achieving equity in health for all people worldwide."[1]

Global Health Diplomacy (GHD). We understand GHD as the "policy shaping processes through which state, non-state and other institutional actors negotiate responses to health challenges or utilize health concepts or mechanisms in policy-shaping and negotiation strategies, to achieve other political, economic or social objectives."[2]

Note: Lee and Smith reproduce this wording and attribute it to an unpublished WHO background paper by Fidler (2009); because that document was not accessible to us, we cite Lee and Smith as the immediate source.[2] For broader overviews of GHD, see Kickbusch et al [3], Kickbusch and Liu,[4] Lee and Smith,[2] and Ruckert et al.[5] Others also position GHD within broader cooperation between state and non-state actors in the international system.[5]

Governance and global health relationships. We differentiate between global governance for health and governance for global health. The former refers to how global governance institutions outside the health sector influence the broad social determinants of health. The latter implies governance arrangements needed to further agreed global health goals.[6] As the difference between domestic and

foreign policy is increasingly artificial, this distinction did not limit our analysis; both are highly interconnected and affect global health.[6]

Health and foreign policy relationships. Foreign policy as health maintains that foreign policy now pursues—and should in the future pursue—health as an end in itself. Health and foreign policy maintains that health's rise on foreign policy agendas indicates that foreign policy is shaping health, not vice versa; health has become another issue with which traditional foreign policy must grapple. Health as foreign policy, on the other hand, is conceptualized differently: its goal is neither health for all nor the balance of power, but the creation of a constructive relationship between health and foreign policy that avoids being utopian, hegemonic, or irrelevant.[7]

Health inequities. Health inequities are systematic, avoidable, and unfair differences in health outcomes observed between populations, between social groups within the same population, or as a gradient across a population ranked by social position.[8]

Globalization. This term has multiple, contested definitions. We use a definition commonly operationalized in global health quantitative measures: "a process of greater integration within the world economy through movements of goods and services, capital, technology and (to a lesser extent) labour, which lead increasingly to economic decisions being influenced by global conditions." [9]

Global South versus Global North. There is no single, agreed definition of these terms. We use "Global South" and "Global North" as broad, relational terms that foreground geopolitical and historical power relations rather than fixed geography.[10]

Text S2: Search strategies:

Web of Science

(TI=((power AND asymmetr* AND global AND health) OR (power AND health AND politics))
OR AB=((power AND asymmetr* AND global AND health) OR (power AND health AND politics))
OR AK=((power AND asymmetr* AND global AND health) OR (power AND health AND politics)))

MEDLINE (Ovid)

((power AND asymmetr* AND global AND health) OR (power AND health AND politics)).ti,ab,kf.

CINAHL (EBSCOhost)

TI(((power AND asymmetr* AND global AND health) OR (power AND health AND politics)))
OR AB(((power AND asymmetr* AND global AND health) OR (power AND health AND politics)))

PsycINFO (EBSCOhost)

TI(((power AND asymmetr* AND global AND health) OR (power AND health AND politics)))

OR AB(((power AND asymmetr* AND global AND health) OR (power AND health AND politics)))
OR KW(((power AND asymmetr* AND global AND health) OR (power AND health AND politics)))

Scopus

TITLE-ABS-KEY((power AND asymmetr* AND global AND health) OR (power AND health AND politics)))

References (Supplementary File 1):

1. Koplan JP, Bond TC, Merson MH, et al. Towards a common definition of global health. *The Lancet.* 2009;373(9679):1993–1995.

2. Lee K, Smith R. What is 'Global Health Diplomacy'? A Conceptual Review. *Global Health Governance.* 2011;5.

3. Kickbusch I, Novotny TE, Drager N, Silberschmidt G, Alcazar S. Global health diplomacy: training across disciplines. *Bulletin of the World Health Organization.* 2007;85:971–973.

4. Kickbusch I, Liu A. Global health diplomacy—reconstructing power and governance. *The Lancet.* 2022;399(10341):2156–2166.

5. Ruckert A, Labonté R, Lencucha R, Runnels V, Gagnon M. Global health diplomacy: a critical review of the literature. *Social science & medicine.* 2016;155:61–72.

6. Lee K, Kamradt-Scott A. The multiple meanings of global health governance: a call for conceptual clarity. *Global Health.* 2014;10:28. doi:10.1186/1744-8603-10-28

7. Fidler DP. Health as foreign policy: between principle and power. *Whitehead J. Dipl. & Int'l Rel.* 2005;6:179.

8. McCartney G, Popham F, McMaster R, Cumbers A. Defining health and health inequalities. *Public Health.* 2019;172:22–30. doi:10.1016/j.puhe.2019.03.023

9. Labonté R, Schrecker T. Globalization and social determinants of health: Introduction and methodological background (part 1 of 3). *Global Health.* 2007;3:5. doi:10.1186/1744-8603-3-5

10. Dados N, Connell R. The Global South. *Contexts.* 2012;11(1):12–13. doi:10.1177/1536504212436479
